# Disentangling what works best for whom in comorbidity

**DOI:** 10.1093/sleep/zsac253

**Published:** 2022-10-15

**Authors:** Nick Glozier, Parisa Vidafar

**Affiliations:** Central Clinical School, Faculty of Medicine and Health, The University of Sydney, Sydney, New South Wales, Australia; Australian Research Council Centre of Excellence for Children and Families over the Life Course, Australia; Central Clinical School, Faculty of Medicine and Health, The University of Sydney, Sydney, New South Wales, Australia; Australian Research Council Centre of Excellence for Children and Families over the Life Course, Australia

There are sufficient randomized controlled trials and systematic reviews of cognitive behavioral therapy for insomnia (CBT-I) [1] and digitally delivered CBT-I [2] that we know both modalities improve insomnia symptoms, and these effects can be long lasting [3]. However, as any casual perusal of the Forest plots in the meta-analyses performed by Zachariae et al. [2]. shows, there is wide variability in both the within and between group effect sizes of different digital CBT-I programs, particularly in symptom scores. This presumably reflects differences in the samples, type of control condition used and, importantly, differential effectiveness of the intervention being evaluated. The paper by Mason and colleagues [4] published in this month’s journal extends our knowledge by moving into an area of research that lots of people talk about but almost never happens; comparative effectiveness research. Mason et al. [4]. compare the efficacy of two digitally delivered cognitive behavioral therapy (CBT) programs, one targeting anxiety, and one targeting insomnia. Both CBT programs comprised four-sessions and were delivered to quite a large number of people (*n* = 120), recruited online, with clinically significant comorbid anxiety, and insomnia symptoms determined via scores on the Insomnia Severity Index (ISI) and the Generalized Anxiety Disorder scale (GAD-7).

Their results show that the CBT-I program is equally effective for treating anxiety symptoms and in the short-term, more efficacious for treating insomnia symptoms [4]. They enhance the paper’s quality by showing that many of the people in this study would meet diagnostic criteria for actual disorders, highlighting potential clinical implications of this result. Should we now treat anxiety with digital CBT-I?

A key lesson from Mason et al. [4]. shows that in anxiety and insomnia, as with many behavioral disorders, we need to do far more comparative efficacy trials, and should be comparing interventions to determine the most efficient treatment. This would seem straightforward with digitally delivered programs. However, as with medication and pharmaceuticals, the business model of such programs means this is unlikely. Network meta-analyses could help determine the most effective and/or efficient treatment, but those of digital CBT-I (and other digital interventions) tend to compare “approaches” rather than programs on the, likely false, assumption that all programs are equal, and the differences assessed being, for example, component attributes such as whether they are supported or not [5], rather than whether one program is inherently better than another.

What is also astonishing are the large effects seen from just four supported sessions in Mason et al. [4]. Are these world-beating digital CBT and CBT-I programs? Should other CBT program developers and providers just switch off their servers, and clinicians go and do something else? What we actually know is that this specific CBT-I program shows a temporary superior efficacy for insomnia symptoms compared to this specific CBT anxiety program in a sample of well-educated people, who show low levels of help seeking (5% in the paper) outside of the online world [4]. Does this matter when we attempt to look at the external validity of the study (i.e. is it generalizable)?Well, the short answer is both yes and no.

First, we have no idea if this comparative superior efficacy is a class effect of all CBT-I interventions, as inferred by the author’s conclusion to this study, or just specific to these two programs. This needs replicating, comparing other programs and using other recruitment strategies. Second, we need to know whether this large effect size exists in samples of people seen in clinics, who use so many resources and represent those with the “burden” referred to in the study by Mason and colleagues [4]. As clinical epidemiology is showing, there is quite a difference in the symptomatic course, and prognosis, of people who are screened and diagnosed from community recruitment compared to people, ostensibly with the same diagnosis, seen in specialist clinics. For instance the short-term insomnia remission rate after four sessions and ninety minutes of online support, determined via scores on the ISI, was 40% in this internet recruited sample [4], yet in a clinically recruited sample of 211 people with insomnia disorder and comorbid psychiatric disorder, much lower remission rates of 26% and 15% were seen after 6 weeks of either face to face clinical psychologist- or physician assistant-delivered manualized and supervised behavioral therapy or zopiclone, respectively [6]. A second 6 weeks of either another therapy or medication was required to achieve the much higher remission rates seen here. In a similar fashion, the effect sizes for CBT-I’s impact on depressive symptoms is larger for internet recruited samples [7] than in people with major depressive disorder under psychiatric care [8]. As such, making inferences about treatment effects across different populations can be fraught.

Conversely, this study by Mason et al. [4]. does provide two important guides. Anxiety symptom rates have been rising, particularly amongst young males, for some years, and were probably exacerbated during the COVID-19 pandemic [9] (although much of the lockdown effect has been transient [10]). Whether more serious forms of anxiety disorder have increased is debatable. Similarly, community rates of insomnia symptoms have also increased, although again, rates of insomnia disorder itself have probably not [11]. Many psychological treatments have moved away from in-person sessions towards telepsychology and/or telehealth, and demand for this delivery method has increased. Efficacious early online treatments could be better implemented into systemwide approaches to stepped care, enabling a significant number of people in the community, (e.g., either those with lower severity or better prognoses) to benefit without waiting to see clinicians or inefficiently use costly resources. Additionally, this should prompt researchers to conduct further evaluation of treatments for sleep disturbance as transdiagnostic interventions to ameliorating psychiatric morbidity, an approach championed by Allison Harvey [12]. This is consonant with a drive to persuade many psychiatrists and psychologists to take sleep disturbance as a serious comorbidity to be the subject of specific treatment rather than just a symptom of a “primary” psychiatric disorder, and one subject to a very recent research call by the Wellcome Trust, one the world’s best endowed medical research funders.
